# Mediating effect of fat mass, lean mass, blood pressure and insulin resistance on the associations of accelerometer-based sedentary time and physical activity with arterial stiffness, carotid IMT and carotid elasticity in 1574 adolescents

**DOI:** 10.1038/s41371-024-00905-6

**Published:** 2024-02-26

**Authors:** Andrew O. Agbaje

**Affiliations:** 1 Institute of Public Health and Clinical Nutrition, School of Medicine, Faculty of Health Sciences, University of Eastern Finland, Kuopio, Finland; 2 Children’s Health and Exercise Research Centre, Department of Public Health and Sports Sciences, Faculty of Health and Life Sciences, University of Exeter, Exeter, UK

**Keywords:** Preventive medicine, Lifestyle modification

## Abstract

This study examined the mediating effect of total body fat mass, lean mass, blood pressure (BP) and insulin resistance on the associations of sedentary time (ST), light physical activity (LPA) and moderate-to-vigorous PA (MVPA) with carotid-femoral pulse wave velocity (cfPWV), carotid intima-media thickness (cIMT) and carotid elasticity in 1574 adolescents from the Avon Longitudinal Study of Parents and Children birth cohort, UK. ST, LPA and MVPA were assessed with ActiGraph accelerometer. ST and LPA were sex-categorised in tertiles as low (reference), moderate and high, while MVPA was categorised as <40 min/day (reference), 40–<60 min/day and ≥60 min/day. cfPWV, cIMT and carotid elasticity were measured with Vicorder and ultrasound. Fat mass and lean mass were assessed with dual-energy X-ray absorptiometry and homeostatic model assessment of insulin resistance (HOMA-IR) was computed. Mediation analyses structural equation models and linear mixed-effect models adjusted for cardiometabolic and lifestyle factors were conducted. Among 1574 adolescents [56.2% female; mean (SD) age 15.4 (0.24) years], 41% males and 17% females accumulated ≥60 min/day of MVPA. Higher ST was associated with lower cIMT partly mediated by lean mass. Higher LPA (*standardized β* = −0.057; [*95% CI* −0.101 to −0.013; *p* = 0.014]) and the highest LPA tertile were associated with lower cfPWV. BP had no significant mediating effect movement behaviour relations with vascular indices. Lean mass partially mediated associations of higher MVPA with higher cIMT (0.012; [0.007–0.002; *p* = 0.001], 25.5% mediation) and higher carotid elasticity (0.025; [0.014–0.039; *p* = 0.001], 28.1% mediation). HOMA-IR mediated the associations of higher MVPA with higher carotid elasticity (7.7% mediation). Engaging in ≥60 min/day of MVPA was associated with higher carotid elasticity. In conclusion, higher LPA was associated with lower arterial stiffness, but higher MVPA was associated with thicker carotid wall explained by higher lean mass.

## Introduction

Arterial stiffness, carotid intima-media thickness (cIMT) and carotid elasticity, measures of arterial function and structure, are surrogates of subclinical arteriosclerosis and atherosclerosis and have been associated with cardiometabolic morbidity and mortality [1–10]. Findings from the associations of objectively measured sedentary (ST) and physical activity (PA) with metabolic markers in less than 18 years olds have been used to formulate PA guidelines but evidence on arterial structure and function are few [11–17]. The paucity of gold-standard measures of arterial structure and function and objectively assessed ST, light PA (LPA) and moderate-to-vigorous PA (MVPA) in a large paediatric population may partly explain the knowledge gap [12–14, 16–18]. Recently, it was reported that accelerometer-measured ST may contribute three times more increase to left ventricular mass when compared with MVPA-related physiologic increase and that higher arterial stiffness may suppress the relationship of higher blood pressure (BP) with higher left ventricular mass [19, 20].

Body composition, BP and metabolic markers are determinants of arterial structure and function in youth, however, whether these factors influence the relationships between movement behaviour and arterial structure and function remains unclear [6, 7, 12, 13, 16–18, 21, 22]. This evidence is critical to planning effective movement behaviour associated vascular health programs in adolescents. Therefore, the present study examined the mediating effect of total body fat mass, trunk fat mass, lean mass, BP and insulin resistance on the associations of ST, LPA and MVPA with carotid-femoral pulse wave velocity (cfPWV), cIMT and carotid elasticity in 1574 adolescents aged 15–17 years from the Avon Longitudinal Study of Parents and Children (ALSPAC), UK birth cohort.

## Methods

Data were from the ALSPAC birth cohort, which investigates factors that influence childhood development and growth. Altogether, pregnant women resident in Avon, UK with expected dates of delivery from 1st April 1991 to 31st December 1992 were invited to take part in the study. The initial number of pregnancies enroled is 14,541, of which there was a total of 14,676 foetuses. When the oldest children were approximately 7 years of age, an attempt was made to bolster the initial sample size with eligible cases who had failed to join the study originally resulting in 14,901 children alive at 1 year of age. Regular clinic visits of the children commenced at 7 years of age and are still ongoing into adulthood. In this study, 1574 participants had who had complete ST, LPA, MVPA at age 15 years and arterial measurements at age 17 years were eligible for analyses. Ethical approval for the study was obtained from the ALSPAC Ethics and Law Committee and the Local Research Ethics Committees. Informed consent for the use of data collected via questionnaires and clinics was obtained from participants following the recommendations of the ALSPAC Ethics and Law Committee at the time [23–25]. Consent for biological samples has been collected in accordance with the Human Tissue Act (2004). Please note that the study website contains details of all the data that is available through a fully searchable data dictionary and variable search tool (http://www.bristol.ac.uk/alspac/researchers/our-data/).

### Physical activity and sedentary time assessment

ST, LPA and MVPA were assessed with ActiGraphTM AM7164 2.2 (LLC, Fort Walton Beach, FL, USA) accelerometer worn on the waist for 7 days at 15-year clinic visits [26–28]. A valid day was defined as providing data for at least 10 h per day (excluding sequences of 10 or more minutes with consecutive zero counts) and children were only included in the analyses if they provided at least 3 valid days of recording (two weekdays and one weekend day). The devices capture movement in terms of acceleration as a combined function of frequency and intensity. Data are recorded as counts that result from summing postfiltered accelerometer values (raw data at 30 Hz) into epoch units. Data were processed using Kinesoft software, version 3.3.75 (Kinesoft), according to established protocol. Activity counts per minute threshold as proposed by Everson were used to calculate the amount of time spent; MVPA, >2296 counts per minute (cpm); for LPA, 100–2296 cpm; and for ST, 0–<100 cpm [6, 22, 29]. The Evenson cutpoint used in stratifying activity threshold has been suggested as the most appropriate cut point for youth having shown the best overall performance across all intensity levels [30, 31]. ST and LPA were grouped in tertiles as low, middle and high tertile categories. MVPA was classified according to PA guideline recommendations of <40 min/day as low, 40–<60 min/day as moderate and ≥60 min/day as high [12]. The low category was the reference category.

### Anthropometry and body composition

Anthropometry (height and weight) of participants at age 15 years was assessed in line with standard protocols and body mass index (BMI) was computed as weight in kilograms per height in metres squared [7, 22]. Body composition (total body fat mass, trunk fat mass and lean mass) was assessed using a dual-energy X-ray absorptiometry scanner at 15 years as previously described [2, 7, 22]. All participants had attained puberty by the 17-year clinic visit assessment using a time (years) to age at peak height velocity objective assessment derived from Superimposition by Translation And Rotation mixed-effects growth curve analysis [22, 32]. The participant’s mother’s socioeconomic status was grouped according to the 1991 British Office of Population and Census Statistics classification [33].

### Vascular phenotype

At age 17 years cfPWV was computed from pressure waveforms obtained using the Vicorder device (Skidmore Medical, Bristol, UK) observing standard protocols as detailed earlier [2, 7, 22]. All measurements were taken independently by one of two trained vascular technicians (inter-observer mean difference 0.2 m/s, SD 0.1) [2, 22]. cIMT from the right and left common carotid arteries at 17 years was assessed by ultrasound using a linear 12 MHz transducer (Vivid7, GE Medical, Chicago, Illinois) [7, 22] Interobserver variability for cIMT was assessed in a separate sample of 25 young adults (coefficient of variation: 4.4 ± 2.2%) [2, 6, 7, 22]. For our analysis, we computed the mean of the average measurement of the right and left common carotid arteries as cIMT. Carotid elasticity was computed as the difference between the baseline (end diastole) and peak (systole) vessel diameter.

### Cardiometabolic and lifestyle factors

Heart rate and systolic and diastolic BP were measured with Omron monitor at age 15 years as previously detailed [7, 22]. Hypertensive systolic BP was categorised as ≥130 mmHg based on clinical guidelines in paediatrics [34]. Using standard protocols, fasting blood samples at age 15 years were collected, spun and frozen at −80 °C and a detailed assessment of fasting glucose, insulin, high-sensitivity C-reactive protein, low-density lipoprotein cholesterol, high-density lipoprotein cholesterol and triglycerides has been reported (coefficient of variation was <5%) [2, 7, 22]. Homeostatic model assessment for insulin resistance (HOMA-IR) was computed from (fasting insulin × fasting glucose/22.5) [35]. Questionnaires to assess smoking behaviour were administered at age 15-year clinic visits. A specific question regarding whether participants smoked in the last 30 days was used as an indicator of current smoking status. At the 17-year clinic visit, participants were briefly asked about their personal and family (mother, father and siblings) medical history such as a history of hypertension, diabetes, high cholesterol and vascular disease [36].

### Statistical analysis

Cohort descriptive characteristics were summarized as means and standard deviation, medians and interquartile ranges, or frequencies and percentages. We explored sex differences using Independent *t* tests, Mann Whitney-U tests, or Chi-square tests for normally distributed, skewed or dichotomous variables, respectively. Multicategory variables were analysed using a one-way analysis of variance. Normality was assessed by histogram curve, quantile-quantile plot and Kolmogorov-Smirnov tests. A logarithmic transformation of skewed variables was conducted and normality was confirmed prior to further analysis.

Mediating path analyses using structural equation models separately examined the mediating role of total body fat mass, trunk fat mass, lean mass, systolic BP, diastolic BP and HOMA-IR on the associations of ST, LPA, or MVPA with each of cfPWV, cIMT and carotid elasticity. Analyses were adjusted for age, sex, the time between exposure and outcome measure, high sensitivity C-reactive protein, heart rate, systolic BP, smoking status, family history of hypertension/diabetes/high cholesterol/vascular disease, socioeconomic status, high-density lipoprotein cholesterol, low-density lipoprotein cholesterol, triglyceride, lean mass or total fat mass, glucose and insulin, with ST, LPA, or MVPA depending on the mediator and predictor. *β* from the mediation analyses are standardized regression coefficients. The path models had three equations per regression analysis: the associations of ST, LPA, or MVPA with total body fat mass, trunk fat mass, lean mass, systolic BP, diastolic BP or HOMA-IR (Equation 1); the associations of total body fat mass, trunk fat mass, lean mass, systolic BP, diastolic BP or HOMA-IR with cfPWV, cIMT and carotid elasticity (Equation 2); and the associations of ST, LPA and MVPA with cfPWV, cIMT and carotid elasticity (Equation 3, total effect) and Equation 3’(direct effect) accounted for the mediating role of total body fat mass, trunk fat mass, lean mass, systolic BP, diastolic BP, or HOMA-IR on the longitudinal associations of ST, LPA and MVPA with cfPWV, cIMT and carotid elasticity. The proportion of mediating or suppressing roles was estimated as the ratio of the difference between Equation 3 and Equation 3’ or the multiplication of Equations 1 and 2 divided by Equation 3 and expressed in percentage. A mediating or indirect role is confirmed when there are statistically significant associations between (a) the predictor and mediator, (b) the predictor and outcome, (c) the mediator and outcome and (d) the longitudinal associations between the predictor and outcome variable was attenuated upon inclusion of the mediator [37]. However, when the magnitude of the association between the predictor and outcome is increased upon inclusion of a third variable, a suppression is confirmed [37]. Path analyses were conducted with 1000 bootstrapped samples.

The separate associations of ST, LPA and MVPA categories with each of cfPWV, cIMT and carotid elasticity were examined using linear mixed-effect models. The optimal model was one with sex and predictor as a factor and a random intercept modelled on the subject level. We selected a random effect variance component type and determined the effect of the predictor on the outcome variables. Whilst the mixed effect model assumes that the data are missing at random and is robust for accounting for missing data at follow-up, we elected to additionally conduct 20 cycles of multiple imputations to account for missing data, that have been shown to have a relative efficiency of over 98% in simulating real data [2, 6, 7, 38, 39]. The analysis strategy was adjusted for sex and the time difference between predictor and outcome measures, age, low-density lipoprotein cholesterol, insulin, triglyceride, high-sensitivity C-reactive protein, high-density lipoprotein cholesterol, heart rate, systolic BP, glucose, fat mass, lean mass, smoking status, family history of hypertension/diabetes/high cholesterol/vascular disease and socioeconomic status with an additional adjustment for ST, LPA, or MVPA depending on the predictor.

Lastly, sex-based linear regression analyses were conducted for the associations of ST, LPA and MVPA with each of cfPWV, cIMT and carotid elasticity, adjusting for the above-listed covariates except sex. Based on the evidence that elevated BP may cause premature cardiac damage in youth [40], analyses were conducted for the associations of ST, LPA and MVPA with each of cfPWV, cIMT and carotid elasticity according to systolic hypertension status, adjusting for the above-listed covariates, except systolic BP. Collinearity diagnoses were performed and results with a variance inflation factor <5 were accepted. Differences and associations with a two-sided *p* value < 0.05 were considered statistically significant, and conclusions were based on effect estimates and their confidence intervals. Sidak correction for potential multiple comparisons was applied. The time difference in years between the exposure measurement at age 15 years and the measurement of vascular outcome at age 17 years was included in the model and controlled. Analyses involving 10% of a sample of 10,000 ALSPAC children at 0.8 statistical power, 0.05 alpha and two-sided *p* value would show a minimum detectable effect size of 0.084 standard deviations if they had relevant exposure for a normally distributed quantitative variable [41]. All statistical analyses were performed using SPSS statistics software, Version 27.0 (IBM Corp, Armonk, NY, USA).

## Results

Females had higher ST, lower LPA and MVPA than males and only 17.4% of females accumulated ≥60 min/day of MVPA compared to 40.1% of males (Table 1). Females had lower cfPWV and carotid elasticity than males, higher total body fat mass, trunk fat mass and insulin resistance and lower lean mass and systolic BP. Other characteristics are described in Table 1.

**Table 1:** Descriptive characteristics of adolescents.

Variables	Male (*n* = 690)	Female *n* = (884)	*P* value
	Mean (SD)	Mean (SD)	
Anthropometry			
Age (years)	15.40 (0.23)	15.41 (0.25)	0.420
Height (m)	1.74 (0.77)	1.65 (0.06)	<0.001
^ *^Weight (kg)	61.6 (12.9)	57.0 (11.8)	<0.001
Ethnicity- White (*n*,%)	616 (96.1)	780 (96.5)	0.675
Attained puberty (*n*, %)	642 (97)	845 (100)	<0.001
Body composition			
^ *^Total fat mass (kg)	8.16 (7.27)	17.08 (8.91)	<0.001
^ *^Trunk fat mass (kg)	3.62 (3.47)	7.69 (4.95)	<0.001
Lean mass (kg)	49.18 (6.76)	36.85 (3.81)	<0.001
^ *^Body mass index, BMI (kg/m^2^)	20.08 (3.38)	20.9 (3.91)	<0.001
Overweight/obese (BMI > 24.9 kg/m^2^ (*n*,%)	58 (8.5)	125 (14.3)	<0.001
Fasting plasma metabolic indices			
High-density lipoprotein (mmol/L)	1.24 (0.27)	1.36 (0.29)	<0.001
Low-density lipoprotein (mmol/L)	1.97 (0.52)	2.19 (0.59)	<0.001
^ *^Triglyceride (mmol/L)	0.70 (0.34)	0.77 (0.39)	<0.001
Glucose (mmol/L)	5.28 (0.38)	5.14 (0.38)	<0.001
^ *^Insulin (mU/L)	7.82 (4.37)	9.73 (5.47)	<0.001
^ *^Homeostatic model assessment for insulin resistance	1.83 (1.15)	2.22 (1.32)	<0.001
^ *^High sensitivity C-reactive protein (mg/L)	0.34 (0.56)	0.38 (0.62)	0.856
Vascular measures			
Heart rate (beat/min)	71 (12)	77 (12)	<0.001
Systolic blood pressure (mmHg)	126 (10)	121 (11)	<0.001
Systolic hypertension (≥130 mmHg)	244 (35.7)	164 (18.7)	<0.001
Diastolic blood pressure (mmHg)	68 (9)	68 (9)	0.264
Arterial measures			
Carotid-femoral pulse wave velocity (m/s)	6.06 (0.72)	5.53 (0.61)	<0.001
Carotid intima-media thickness (mm)	0.48 (0.04)	0.47 (0.42)	<0.001
Carotid vessel elasticity (mm)	1.00 (0.18)	0.89 (0.15)	<0.001
Physical activity profile			
Sedentary time (min/day)	461 (87)	483 (81)	<0.001
Light physical activity (min/day)	290 (69)	272 (61)	<0.001
MVPA (min/day)	56 (31)	40 (23)	<0.001
MVPA ≥ 60 min/day (n,%)	277 (40.1)	154 (17.4)	<0.001
Lifestyle factors			
Smoked cigarette in last 30 days (*n*,%)	45 (6.7)	109 (12.5)	<0.001
Family history of H-D-C-V (*n*,%)	200 (29)	253 (28.7)	0.911
Socio-economic status (*n*, %)			0.807
Professional	29 (8.6)	23 (5.8)	
Managerial and technical	131 (39.0)	159 (40.4)	
Skilled non-manual	106 (31.5)	139 (35.3)	
Skilled manual	4 (1.2)	7 (1.8)	
Partly skilled	54 (16.1)	57 (14.5)	
Unskilled	12 (3.6)	9 (2.3)	

The values are means (standard deviations), ^*^median (interquartile range) and frequency and percentage. Differences between sexes were tested using Student’s *t* test for normally distributed continuous variables, Mann–Whitney U test for skewed continuous variables, Chi-square test for dichotomous variables and one-way ANOVA for multicategorical variable. A two-sided *P* value < 0.05 is considered statistically significant. *P* value for sex differences.

*H-D-C-V* hypertension, diabetes, high cholesterol and vascular disease, *MVPA* moderate-to-vigorous physical activity.

### Sedentary time with cfPWV, cIMT and carotid elasticity

Higher ST was consistently associated with lower cIMT irrespective of the mediators (Tables 2–7). Total body fat mass, trunk fat mass, systolic BP, diastolic BP and insulin resistance, had no significant mediating effect on any associations between ST and arterial measures (Tables 2, 3, 5–7). Lean mass had a partial mediating effect (11.8%) on the associations of higher ST with lower cIMT (Table 4). Middle ST tertile was associated with higher carotid elasticity and there are no sex differences in ST relations with arterial measures (Tables 8, 9). There were no statistically significant associations between higher ST and vascular indices in participants with either normal systolic BP or systolic hypertension (Table 10).

**Table 2:** Mediating or suppressing role of total body fat mass on the associations of movement behaviour with arterial measures.

Mediator: Total fat mass							
(*N* = 1574)	Total effect		Direct effect		Indirect effect		Mediation (%)
Predictors	*β* (95% CI)	*p* value	*β* (95% CI)	*p* value	*β* (95% CI)	*p* value	
Carotid-femoral pulse wave velocity							
Sedentary time	0.029 (−0.018–0.076)	0.212	0.034 (−0.014–0.081)	0.152	−0.005 (−0.013–0.002)	0.168	17.2
Light PA	−0.057 (−0.101 to −0.013)	**0.014**	−0.058 (−0.103 to −0.011)	**0.011**	0.001 (−0.005–0.008)	0.641	1.75
MVPA	0.003 (−0.041–0.052)	0.898	0.003 (−0.042–0.053)	0.893	0.000 (−0.007–0.006)	0.993	0
Carotid intima-media thickness							
Sedentary time	−0.054 (−0.102 to −0.006)	**0.028**	−0.052 (−0.101 to −0.004)	**0.033**	−0.002 (−0.006–0.001)	0.120	3.7
Light PA	0.038 (−0.015–0.093)	0.120	0.038 (−0.016–0.091)	0.137	0.000 (−0.002–0.004)	0.567	0
MVPA	0.038 (−0.010–0.095)	0.132	0.038 (−0.010–0.096)	0.128	0.000 (−0.003–0.002)	0.960	0
Carotid wall elasticity							
Sedentary time	−0.018 (−0.065–0.028)	0.401	−0.014 (−0.063–0.030)	0.469	−0.004 (−0.009–0.002)	0.173	22.2
Light PA	0.053 (0.002–0.105)	**0.043**	0.052 (0.000–0.105)	0.052	0.001 (−0.004–0.006)	0.641	1.9
MVPA	0.073 (0.024–0.118)	**0.007**	0.072 (0.026–0.118)	**0.006**	0.000 (−0.005–0.004)	0.989	0

Bold values indicate statistical significance *p* < 0.05.

Mediation structural equation model was adjusted for age, sex, time between exposure and outcome measure, high sensitivity C-reactive protein, heart rate, systolic blood pressure, smoking status, family history of hypertension/diabetes/high cholesterol/vascular disease, socioeconomic status, lean mass, high-density lipoprotein cholesterol, low-density lipoprotein cholesterol, triglyceride, glucose and insulin, with sedentary time, light physical activity (light PA), or moderate-to-vigorous physical activity (MVPA) depending on the predictor. *β* is standardized regression coefficient. *p* value < 0.05 were considered statistically significant.

**Table 3:** Mediating or suppressing role of trunk fat mass on the associations of movement behaviour with arterial measures.

Mediator: Trunk fat mass							
(*N* = 1574)	Total effect		Direct effect		Indirect effect		Mediation (%)
Predictors	*β* (95% CI)	*p* value	*β* (95% CI)	*p* value	*β* (95% CI)	*p* value	
Carotid-femoral pulse wave velocity							
Sedentary time	0.029 (−0.018–0.078)	0.207	0.033 (−0.015–0.081)	0.155	−0.004 (−0.011–0.003)	0.219	13.8
Light PA	−0.056 (−0.100 to −0.010)	**0.016**	−0.059 (−0.103 to −0.011)	**0.011**	0.002 (−0.004–0.009)	0.356	3.6
MVPA	0.004 (−0.041–0.052)	0.869	0.004 (−0.041–0.053)	0.866	0.000 (−0.007–0.006)	0.967	0
Carotid intima-media thickness							
Sedentary time	−0.054 (−0.101 to −0.006)	**0.029**	−0.052 (−0.100 to −0.004)	**0.034**	−0.002 (−0.006–0.001)	0.195	3.7
Light PA	0.038 (−0.016–0.093)	0.128	0.037 (−0.017–0.091)	0.142	0.001 (−0.001–0.004)	0.303	2.6
MVPA	0.038 (−0.010–0.095)	0.130	0.038 (−0.010–0.096)	0.124	0.000 (−0.003–0.002)	0.977	0
Carotid wall elasticity							
Sedentary time	−0.018 (−0.065–0.028)	0.418	−0.015 (−0.063–0.031)	0.482	−0.003 (−0.009–0.002)	0.215	16.6
Light PA	0.053 (0.003–0.106)	**0.042**	0.051 (−0.001–0.104)	0.057	0.002 (−0.003–0.007)	0.371	3.8
MVPA	0.073 (0.025–0.119)	**0.006**	0.073 (0.026–0.120)	**0.006**	0.000 (−0.005–0.004)	0.969	0

Bold values indicate statistical significance *p* < 0.05.

Mediation structural equation model was adjusted for age, sex, time between exposure and outcome measure, high sensitivity C-reactive protein, heart rate, systolic blood pressure, smoking status, family history of hypertension/diabetes/high cholesterol/vascular disease, socioeconomic status, lean mass, high-density lipoprotein cholesterol, low-density lipoprotein cholesterol, triglyceride, glucose and insulin, with sedentary time, light physical activity (light PA), or moderate-to-vigorous physical activity (MVPA) depending on the predictor. *β* is standardized regression coefficient. *p* value < 0.05 were considered statistically significant.

**Table 4:** Mediating or suppressing role of lean mass on the associations of movement behaviour with arterial measures.

Mediator: Lean mass							
(*N* = 1574)	Total effect		Direct effect		Indirect effect		Mediation (%)
Predictors	*β* (95% CI)	*p* value	*β* (95% CI)	*p* value	*β* (95% CI)	*p* value	
Carotid-femoral pulse wave velocity							
Sedentary time	0.035 (−0.008–0.085)	0.098	0.023 (−0.024–0.067)	0.297	0.012 (0.000–0.025)	0.052	34.3
Light PA	−0.042 (−0.085–0.003)	0.065	−0.050 (−0.089 to −0.001)	**0.042**	0.008 (−0.004–0.019)	0.229	19.1
MVPA	0.024 (−0.018–0.068)	0.274	−0.001 (−0.043–0.046)	0.957	0.025 (0.015–0.037)	0.001	104.2
Carotid intima-media thickness							
Sedentary time	−0.051 (−0.099 to −0.004)	**0.037**	−0.057 (−0.105 to −0.010)	**0.020**	0.006 (0.000–0.013)	**0.041**	**11.8**
Light PA	0.045 (−0.007–0.097)	0.081	0.041 (−0.013–0.093)	0.119	0.004 (−0.002–0.010)	0.206	8.9
MVPA	0.047 (0.000–0.103)	**0.047**	0.034 (−0.013–0.090)	0.152	0.012 (0.007–0.020)	**0.001**	**25.5**
Carotid wall elasticity							
Sedentary time	−0.011 (−0.054–0.035)	0.607	−0.023 (−0.068–0.020)	0.249	0.012 (0.000–0.026)	0.046	109.1
Light PA	0.065 (0.014–0.114)	**0.012**	0.057 (0.007–0.107)	**0.027**	0.008 (−0.004–0.019)	0.229	12.3
MVPA	0.089 (0.044–0.133)	**0.002**	0.064 (0.018–0.106)	**0.011**	0.025 (0.014–0.039)	**0.001**	**28.1**

Bold values indicate statistical significance *p* < 0.05.

Mediation structural equation model was adjusted for age, sex, time between exposure and outcome measure, high sensitivity C-reactive protein, heart rate, systolic blood pressure, smoking status, family history of hypertension/diabetes/high cholesterol/vascular disease, socioeconomic status, total body fat mass, high-density lipoprotein cholesterol, low-density lipoprotein cholesterol, triglyceride, glucose and insulin, with sedentary time, light physical activity (light PA), or moderate-to-vigorous physical activity (MVPA) depending on the predictor. *β* is standardized regression coefficient. *p* value < 0.05 were considered statistically significant.

**Table 5:** Mediating or suppressing role of systolic blood pressure on the associations of movement behaviour with arterial measures.

Mediator: Systolic blood pressure							
(*N* = 1574)	Total effect		Direct effect		Indirect effect		Mediation (%)
Predictors	*β* (95% CI)	*p* value	*β* (95% CI)	*p* value	*β* (95% CI)	*p* value	
Carotid-femoral pulse wave velocity							
Sedentary time	0.021 (−0.027–0.068)	0.365	0.022 (−0.026–0.069)	0.345	−0.001 (−0.013–0.009)	0.794	4.8
Light PA	−0.046 (−0.091–0.003)	0.063	−0.043 (−0.091–0.002)	0.066	−0.002 (−0.013–0.009)	0.663	4.3
MVPA	0.022 (−0.023–0.069)	0.331	0.025 (−0.019–0.074)	0.254	−0.003 (−0.013–0.006)	0.445	13.6
Carotid intima-media thickness							
Sedentary time	−0.057 (−0.106 to −0.009)	**0.019**	−0.057 (−0.105 to −0.008)	**0.021**	0.000 (−0.006–0.004)	0.751	0
Light PA	0.042 (−0.011–0.097)	0.090	0.043 (−0.007–0.098)	0.082	−0.001 (−0.006–0.003)	0.597	2.4
MVPA	0.045 (−0.003–0.101)	0.065	0.046 (0.000–0.104)	0.052	−0.001 (−0.007–0.002)	0.405	2.2
Carotid wall elasticity							
Sedentary time	−0.023 (−0.071–0.022)	0.280	−0.022 (−0.071–0.022)	0.292	−0.001 (−0.009–0.006)	0.752	4.3
Light PA	0.060 (0.007–0.112)	**0.025**	0.061 (0.011–0.115)	**0.020**	−0.002 (−0.009–0.005)	0.606	3.3
MVPA	0.086 (0.039–0.132)	**0.001**	0.088 (0.040–0.133)	**0.002**	−0.002 (−0.010–0.003)	0.420	2.3

Bold values indicate statistical significance *p* < 0.05.

Mediation structural equation model was adjusted for age, sex, time between exposure and outcome measure, high sensitivity C-reactive protein, heart rate, total body fat mass, lean mass, smoking status, family history of hypertension/diabetes/high cholesterol/vascular disease, socioeconomic status, high-density lipoprotein cholesterol, low-density lipoprotein cholesterol, triglyceride, glucose and insulin, with sedentary time, light physical activity (light PA), or moderate-to-vigorous physical activity (MVPA) depending on the predictor. *β* is standardized regression coefficient. *p* value < 0.05 were considered statistically significant.

**Table 6:** Mediating or suppressing role of diastolic blood pressure on the associations of movement behaviour with arterial measures.

Mediator: Diastolic blood pressure							
(*N* = 1574)	Total effect		Direct effect		Indirect effect		Mediation (%)
Predictors	*β* (95% CI)	*p* value	*β* (95% CI)	*p* value	*β* (95% CI)	*p* value	
Carotid-femoral pulse wave velocity							
Sedentary time	0.023 (−0.026–0.069)	0.367	0.016 (−0.031–0.063)	0.447	0.006 (−0.002–0.016)	0.107	26.1
Light PA	−0.044 (−0.089–0.003)	0.081	−0.042 (−0.086–0.006)	0.087	−0.002 (−0.013–0.009)	0.791	4.5
MVPA	0.019 (−0.026–0.067)	0.407	0.025 (−0.021–0.074)	0.295	−0.007 (−0.017–0.002)	0.090	36.8
Carotid intima−media thickness							
Sedentary time	−0.055 (−0.103 to −0.004)	**0.029**	−0.053 (−0.104 to −0.004)	**0.029**	−0.001 (−0.011–0.007)	0.170	1.8
Light PA	0.041 (−0.012–0.097)	0.098	0.040 (−0.013–0.096)	0.107	0.000 (−0.001–0.005)	0.518	0
MVPA	0.043 (−0.005–0.100)	0.089	0.041 (−0.008–0.099)	0.107	0.001 (−0.001–0.006)	0.152	2.3
Carotid wall elasticity							
Sedentary time	−0.017 (−0.065–0.028)	0.419	−0.011 (−0.060–0.038)	0.617	−0.006 (−0.016–0.001)	0.107	35.3
Light PA	0.055 (0.005–0.108)	**0.037**	0.054 (0.004–0.104)	**0.039**	0.001 (−0.007–0.011)	0.748	1.8
MVPA	0.081 (0.034–0.129)	**0.004**	0.074 (0.027–0.120)	**0.003**	0.006 (-0.002–0.015)	0.095	7.4

Bold values indicate statistical significance *p* < 0.05.

Mediation structural equation model was adjusted for age, sex, time between exposure and outcome measure, high sensitivity C-reactive protein, heart rate, total body fat mass, lean mass, smoking status, family history of hypertension/diabetes/high cholesterol/vascular disease, socioeconomic status, high-density lipoprotein cholesterol, low-density lipoprotein cholesterol, triglyceride, glucose and insulin, with sedentary time, light physical activity (light PA), or moderate-to-vigorous physical activity (MVPA) depending on the predictor. *β* is standardized regression coefficient. *p* value < 0.05 were considered statistically significant.

**Table 7:** Mediating or suppressing role of insulin resistance on the associations of movement behaviour with arterial measures.

Mediator: Insulin Resistance							
(*N* = 1574)	Total effect		Direct effect		Indirect effect		Mediation (%)
Predictors	*β* (95% CI)	*p* value	*β* (95% CI)	*p* value	*β* (95% CI)	*p* value	
Carotid-femoral pulse wave velocity							
Sedentary time	0.030 (−0.018–0.078)	0.203	0.026 (−0.023–0.073)	0.287	0.004 (0.000–0.009)	0.043	13.3
Light PA	−0.050 (−0.095 to −0.003)	**0.032**	−0.050 (−0.097 −0.002)	**0.031**	−0.001 (−0.006–0.004)	0.684	2.0
MVPA	0.013 (−0.033–0.062)	0.555	0.007 (−0.038–0.057)	0.724	0.005 (0.001–0.012)	0.003	38.5
Carotid intima-media thickness							
Sedentary time	−0.053 (−0.103 to −0.004)	**0.027**	−0.055 (−0.103 to −0.007)	**0.027**	0.002 (0.000–0.007)	0.080	3.8
Light PA	0.040 (−0.013–0.095)	0.106	0.041 (−0.013–0.096)	0.104	0.000 (−0.004–0.002)	0.546	0
MVPA	0.041 (−0.007–0.097)	0.104	0.039 (−0.010–0.095)	0.131	0.002 (0.000–0.009)	0.081	4.9
Carotid wall elasticity							
Sedentary time	−0.015 (−0.065–0.030)	0.461	−0.020 (−0.069–0.026)	0.346	0.005 (0.000–0.011)	0.041	33.3
Light PA	0.056 (0.004–0.109)	**0.037**	0.057 (0.005–0.111)	**0.031**	−0.001 (−0.007–0.004)	0.674	1.8
MVPA	0.078 (0.030–0.123)	**0.004**	0.072 (0.025–0.117)	**0.006**	0.006 (0.002–0.013)	**0.003**	**7.7**

Bold values indicate statistical significance *p* < 0.05.

Mediation structural equation model was adjusted for age, sex, time between exposure and outcome measure, high sensitivity C-reactive protein, heart rate, systolic BP, smoking status, family history of hypertension/diabetes/high cholesterol/vascular disease, socioeconomic status, total body fat mass, lean mass, high-density lipoprotein cholesterol, low-density lipoprotein cholesterol, triglyceride, with sedentary time, light physical activity (light PA), or moderate-to-vigorous physical activity (MVPA) depending on the predictor. *β* is standardized regression coefficient. *p* value < 0.05 were considered statistically significant. Insulin resistance was calculated from the homeostatic model assessment of insulin resistance (HOMA-IR) from (fasting plasma insulin × fasting plasma glucose/22.5).

**Table 8:** Sedentary time and physical activity categories in relation to arterial structure and function.

*N* = 1574	Carotid-femoral PWV		Carotid intima-media thickness		Carotid wall elasticity	
Predictor	*β*	*P* value	*β*	*P* value	*β*	*P* value
Sedentary time (lowest tertile)	*Reference*		*Reference*		*Reference*	
Moderate tertile	0.052	0.203	−0.001	0.680	0.022	**0.030**
High tertile	0.039	0.434	−0.004	0.207	0.017	0.163
Light PA (lowest tertile)	*Reference*		*Reference*		*Reference*	
Moderate tertile	−0.012	0.768	0.001	0.711	0.026	**0.010**
High tertile	−0.107	**0.018**	−0.0001	0.961	0.021	0.060
MVPA < 40 min/day	*Reference*		*Reference*		*Reference*	
40–<60 min/day	−0.013	0.749	0.001	0.447	0.001	0.402
≥60 min/day	0.042	0.346	−0.002	0.738	0.024	**0.027**

Bold values indicate statistical significance *p* < 0.05.

Analyses were conducted using linear mixed-effect model and adjusted for age, sex, time difference in years between measure of predictor and outcome, high-density lipoprotein cholesterol, low-density lipoprotein cholesterol, triglyceride, glucose, insulin, heart rate, systolic BP, high-sensitivity C-reactive protein, lean mass, total body fat mass, smoking status, family history of cardiovascular and metabolic disease, socio-economic status and sedentary time, light physical activity or moderate-to-vigorous (MV) physical activity, depending on the predictor. *β* is effect estimate derived from unstandardized linear mixed-effect model. Statistically significant associations were determined by two-sided *p* value < 0.05. Carotid wall elasticity or distensibility is the difference between peak (systole) carotid vessel diameter and baseline (end-diastole) carotid vessel diameter. Missing covariates were accounted for using 20 cycles of multiple imputation.

*PA* physical activity, *PWV* pulse wave velocity.

**Table 9:** Sedentary time and physical activity in relation to arterial structure and function based on sex category.

*N* = 1574	Carotid-femoral PWV		Carotid intima-media thickness		Carotid wall elasticity	
Predictor	*β*	*P* value	*β*	*P* value	*β*	*P* value
Male						
Sedentary time	0.017	0.609	−0.002	0.355	0.011	0.172
Light PA	−0.027	0.407	0.001	0.444	0.013	0.112
Moderate to vigorous PA	0.052	0.099	0.002	0.211	0.019	**0.013**
Female						
Sedentary time	0.003	0.912	−0.002	0.276	0.002	0.716
Light PA	−0.048	**0.044**	−0.001	0.619	0.008	0.155
Moderate to vigorous PA	−0.001	0.949	−0.001	0.685	0.003	0.513

Bold values indicate statistical significance *p* < 0.05.

Analyses were conducted using linear regression model and adjusted for age, time difference in years between measure of predictor and outcome, high-density lipoprotein cholesterol, low-density lipoprotein cholesterol, triglyceride, glucose, insulin, heart rate, systolic blood pressure, high-sensitivity C-reactive protein, lean mass, total body fat mass, smoking status, family history of cardiovascular and metabolic disease, socio-economic status and sedentary time, light physical activity or moderate physical activity depending on predictor. *β* is standardized regression coefficient. Statistically significant associations were determined by two-sided *p* value < 0.05. Carotid wall elasticity or distensibility is the difference between peak (systole) carotid vessel diameter and baseline (end diastole) carotid vessel diameter. Missing covariates were accounted for using 20 cycles of multiple imputations.

*PA* physical activity, *PWV* pulse wave velocity.

**Table 10:** Sedentary time and physical activity in relation to arterial structure and function based on systolic hypertension ( ≥130 mmHg) category.

	Carotid-femoral PWV		Carotid intima-media thickness		Carotid wall elasticity	
Predictor	*β* (95% CI)	*P* value	*Β* (95% CI)	*P* value	*Β* (95% CI)	*P* value
Normotensive systolic BP (*n* = 1130)						
Sedentary time	−0.007 (−0.072–0.057)	0.821	−0.002 (−0.104–0.033)	0.307	0.043 (−0.022–0.109)	0.197
Light PA	−0.064 (−0.127 to −0.001)	**0.046**	0.001 (−0.067–0.068)	0.992	0.078 (0.014–0.143)	**0.018**
Moderate to vigorous PA	0.028 (−0.033–0.090)	0.366	0.042 (−0.024–0.107)	0.211	0.039 (−0.024–0.102)	0.224
Hypertensive systolic BP of ≥130 mmHg (*n* = 429)						
Sedentary time	0.057 (−0.052–0.167)	0.304	−0.054 (−0.168–0.060)	0.355	0.029 (−0.075–0.132)	0.590
Light PA	−0.022 (−0.129–0.085)	0.689	0.034 (−0.079–0.146)	0.558	0.034 (−0.070–0.138)	0.520
Moderate to vigorous PA	0.047 (−0.056–0.150)	0.371	−0.023 (−0.130–0.084)	0.674	0.081 (−0.016–0.178)	0.101

Bold values indicate statistical significance *p* < 0.05.

Analyses were conducted using linear regression model and adjusted for age, sex, time difference in years between measure of predictor and outcome, high-density lipoprotein cholesterol, low-density lipoprotein cholesterol, triglyceride, glucose, insulin, heart rate, high-sensitivity C-reactive protein, lean mass, total body fat mass, smoking status, family history of cardiovascular and metabolic disease, socio-economic status and sedentary time, light physical activity or moderate physical activity depending on predictor. *β* is standardized regression coefficient; CI, confidence interval. Statistically significant associations were determined by two-sided *p* value < 0.05. Carotid wall elasticity or distensibility is the difference between peak (systole) carotid vessel diameter and baseline (end diastole) carotid vessel diameter. Missing covariates were accounted for using 20 cycles of multiple imputations.

*PA* physical activity, *PWV* pulse wave velocity.

### Light physical activity with cfPWV, cIMT and carotid elasticity

Higher LPA was consistently associated with lower cfPWV and higher carotid elasticity irrespective of the mediators (Tables 2–7). Total body fat mass, trunk fat mass, lean mass, systolic BP, diastolic BP and insulin resistance had no significant mediating effect on any associations between LPA and arterial measures (Tables 2–7). The highest LPA tertile was associated with lower cfPWV while the moderate LPA tertile was associated with higher carotid elasticity (Table 8). Higher LPA was associated with lower cfPWV in females but not in males (Table 9). Higher LPA was associated with lower cfPWV and higher carotid elasticity in participants with normotensive systolic BP. There were no significant associations between LPA and vascular indices in participants with systolic hypertension (Table 10).

### Moderate-to-vigorous physical activity with cfPWV, cIMT and carotid elasticity

Higher MVPA was consistently associated with higher carotid elasticity irrespective of the mediators (Tables 2–7). Higher MVPA was also associated with higher cIMT when lean mass was the mediator (Table 4). Total body fat mass, trunk fat mass, systolic BP and diastolic BP had no significant mediating effect on any associations between MVPA and arterial measures (Tables 2, 3, 5 and 6). Lean mass partially mediated the associations of higher MVPA with higher cIMT (25.5% mediation effect) and carotid elasticity (28.1% mediation effect). MVPA category of ≥60 min/day was associated with higher carotid elasticity (Table 8). Higher MVPA was associated with higher carotid elasticity in males but not in females (Table 9). There were no statistically significant associations between higher MVPA and vascular indices in participants with either normal systolic BP or systolic hypertension (Table 10).

## Discussion

This study in a large cohort of apparently healthy adolescents examined the role of objectively assessed body composition, systolic and diastolic BP and insulin resistance on the relationships between movement behaviours and arterial functional and structural measures. It was observed that higher ST was paradoxically associated with lower cIMT, higher LPA was associated with lower cfPWV while higher MVPA was associated with higher carotid elasticity and paradoxically with higher cIMT. Total body fat mass, trunk fat mass and systolic and diastolic BP had no mediating role in these relationships. However, lean mass partially mediated the associations of ST and MVPA with arterial measures, whereas insulin resistance partly mediated the associations of MVPA with arterial measures. Together, these findings suggest explanatory pathways through which movement behaviours exert their influence on arterial function and structure in adolescence.

### Sedentary time with cfPWV, cIMT and carotid elasticity

Objectively measured ST has been associated with worse metabolic and cardiac profiles and higher arterial stiffness in children, adolescents and young adults [11, 12, 15, 19, 27, 28]. In a Brazilian cohort of 1259 young adults aged 30 years, the highest ST quartile was independently associated with higher cfPWV [15]. The Brazilian study also examined the mediating role of BMI, waist circumference and systolic and diastolic BP on the relationships between ST and cfPWV and found that waist circumference had an 18% mediating effect, and diastolic BP had a 27% mediating effect on the relationship [15]. However, the study could not assess the mediating role of lean mass due to the surrogate assessment of body composition employed [15]. The authors did not examine the mediating role of insulin resistance which has been associated with arterial stiffness in this age group [2, 3, 8].

In the present study of 1574 adolescents with a gold-standard measure of body composition, ST either as a continuous or categorical variable was not associated with cfPWV while trunk fat mass and diastolic BP had no mediating effect. The contrasting findings may be related to the significant difference in participants’ age (~15 years) between the Brazilian study and the present study since age is a strong predictor of arterial stiffness [1, 3, 8, 15]. Other reasons are that 58% of the Brazilian study cohort were overweight and obese compared to 11% of participants in the present study [15]. Moreover, the highest ST quartile in the Brazilian study spent 12–16 h per day sedentary, compared to <9 h of ST among adolescents at the highest ST quartile in the present study [15]. A Swedish study of 658 young adults aged 22 years found that ST was not associated with cfPWV which is consistent with the present study [16]. The Brazilian and Swedish studies did not examine the association of ST with measures of cIMT, a surrogate measure of subclinical atherosclerosis and carotid elasticity [5–7, 15, 16]. A recent study conducted among 102 Estonian 12-year-old boys reported that ST was not associated with cfPWV and cIMT measured 6 years later [17]. Paradoxically, in the present study, it was observed that higher ST was associated with lower cIMT, and middle ST tertile was associated with higher carotid elasticity. The paradoxical nature of ST was reported recently such as the relationships of increased ST with increased lean mass and cardiorespiratory fitness [27, 42]. Since increased lean mass drives vascular remodeling, increased ST through increased lean mass might influence vascular architecture [22]. Nonetheless, longitudinal and mechanistic studies on the relationships between ST and vascular measures are warranted in the paediatric population [12, 18].

### Light physical activity with cfPWV, cIMT and carotid elasticity

Higher cfPWV independently predicts cardiometabolic morbidities in youth and mortality in adults [1–4, 7, 8, 21]. Studies on the objective measure of LPA in relation to gold-standard arterial measures in the young are few [16, 43]. A Swedish study of 658 young adults and another study that included 126 children aged 12 years from South Wales reported that LPA was not associated with cfPWV [16, 43]. The present study, which has a 3–15-fold larger population size than the previous studies revealed that higher LPA and highest LPA tertile were associated with lower cfPWV and that middle or moderate tertile category of LPA was associated with higher carotid elasticity [16, 43]. These findings are of public health significance in the adolescent population who are unable to engage in vigorous exercise due to morbidities or lack of interest [1, 8, 12]. LPA was not associated with cIMT and neither body composition, BP nor insulin resistance mediated the relationships of LPA with arterial measures. The finding also suggests that the LPA may likely contribute to arterial functional (cfPWV) rather than structural (cIMT) alterations. Although engaging in LPA has not been shown to increase lean mass in the young population, it may potentially lower BP which may enhance arterial function but more mechanistic studies are warranted [27, 44]. The benefit of LPA has been reported in several adult studies, such as doubling the time spent in LPA was associated with a 29% reduction in mortality [45]. In this cohort, higher LPA was associated with lower cfPWV and higher carotid elasticity in participants with normal systolic BP, but no associations were found among participants with systolic hypertension. The findings among adolescents with systolic hypertension could be due to low statistical power to detect a meaningful effect since only one-quarter of the adolescents had systolic BP ≥130 mmHg. Nonetheless, BP had no significant mediating effect on the relationship between LPA and vascular indices.

### Moderate-to-vigorous physical activity with cfPWV, cIMT and carotid elasticity

Recently, a one-year MVPA randomized clinical trial in 203 young adults aged 22 years with hypertension reported no effect on cfPWV [46]. Moreover, an 8-month MVPA randomized clinical trial in 175 overweight and obese 10-year-old children did not alter cfPWV [47]. Among 12-year-old 126 children from South Wales, MVPA was not associated with cfPWV [43]. In consonance with the previous studies [43, 46, 47], the present study found that MVPA was not associated with cfPWV. However, in the Brazilian young adult study, higher MVPA was associated with lower cfPWV and both waist circumference and diastolic BP had 44% and 33% mediating effects, respectively [15]. The contrast in results may be due to 5-fold more participants with overweight and obese compared to the present study, in addition to the 15-year difference in participant age [15]. Participants who were overweight and obese had higher cfPWV compared to those with normal weight [22]. In the present study, neither total body fat mass, trunk fat mass, systolic BP nor diastolic BP had a mediating effect on the associations of MVPA with arterial measures. Higher MVPA was associated with higher cIMT only when lean mass partially mediated the relationships. In the presence of other mediating factors, MVPA was not associated with cIMT. Higher MVPA and MVPA category of ≥60 min/day were consistently and independently associated with carotid elasticity, irrespective of the mediating factors, with lean mass and insulin resistance partly mediating the relationships. The emerging physiologic role of lean mass in arterial function and structure due to the availability of gold-standard measures of body composition has improved the understanding of vascular adaptations in the paediatric population which was previously misinterpreted as the adverse consequence of high BMI [7, 22, 48, 49].

Nearly one-third of the effect of MVPA on carotid elasticity was effected via a higher lean mass. Lean mass may have bi-directional relationships with arterial structure and function, thus a muscle mass increase-related intervention may improve arterial health while engaging in ≥60 min/day of MVPA [7, 12, 27]. Increasing MVPA levels in females relative to males may be crucial to the carotid elasticity benefit of MVPA. The paradoxical relationships between higher MVPA and higher cIMT, when lean mass is a mediator, may be related to chronic vascular remodeling due to repetitive wall strain and stress from increased cardiac output, heart rate, BP and atherogenesis [1, 7, 10, 27, 50–52]. Prospective studies in youth are warranted to examine whether persistent high MVPA levels retain paradoxical relationships with arterial structure since the evidence would be important for recommending MVPA in paediatric populations.

### Strength and limitations

A large adolescent population with extensive gold-standard measures of relevant variables enabled high statistical powered comprehensive analyses of the relationships between movement behaviours and arterial function and structure. A few limitations include under or over-estimation of movement behaviours due to likely overlap in distinguishing between LPA and ST while using 60 s epoch during accelerometer measures. The lack of movement behaviour measures at age 17 years when the arterial assessments were conducted although the time difference in measurement was accounted for in the analyses. The transition between age 15–17 years is a crucial time for metabolic alterations such as the bi-directional relationship between fat mass and insulin resistance begins at age 17 years and a time when PA declines due to changes in socio-economic and lifestyle factors [53]. This period between ages 15–17 years has also been identified as a critical time to reverse dyslipidaemia-related subclinical atherosclerosis in the young population [6, 54]. Thus, a negative PA lifestyle habit might have been reinforced between age 15–17 years which could be deleterious to vascular health. Representing movement behaviours over just 30 h from 7 days may lead to relatively low reliability of the measurement, however, accelerometer-based PA measure overcomes the bias inherent in questionnaire-accessed PA. There is low evidence for causal inference due to the observational study design. Lastly, nearly all study participants are Caucasians which limits the generalisability of the findings to other ethnicities.

## Conclusion

Higher ST was paradoxically associated with lower cIMT and had no relationships with cfPWV. Middle tertile ST was associated with higher carotid elasticity. Higher LPA and highest LPA tertile were significantly associated with lower cfPWV. Middle tertile LPA was associated with higher carotid elasticity. Higher MVPA and ≥60 min/day of MVPA were associated with higher carotid elasticity and paradoxically with higher cIMT but not with cfPWV. Lean mass was the strongest mediating factor in the relationships of higher ST with lower cIMT and higher MVPA with higher cIMT and carotid elasticity. Insulin resistance partly mediated the relationships of higher MVPA with higher carotid elasticity. Total body fat mass, trunk fat mass, systolic BP and diastolic BP had no significant mediating effects in the relationship between movement behaviour and arterial function and structure. The effective prevention of subclinical arteriosclerosis and atherosclerosis in mid- through late adolescence may focus on improving LPA and MVPA and increasing muscle mass, especially in females.

## Summary

### What is known about topic

- Physical activity is beneficial for metabolic health but its role on vascular health remains controversial.
- Sedentary behaviour has been associated with the risk of obesity, dyslipidemia, inflammation and cardiac hypertrophy but its role in vascular health requires further research.

### What this study adds

- Light physical activity was associated with better vascular health, i.e. lower arterial stiffness.
- Moderate-to-vigorous physical activity was associated with higher carotid thickness partly explained by higher lean mass.

## Data Availability

The informed consent obtained from ALSPAC participants does not allow the data to be made freely available through any third-party maintained public repository. However, data used for this submission can be made available on request to the ALSPAC Executive. The ALSPAC data management plan describes in detail the policy regarding data sharing, which is through a system of managed open access. Full instructions for applying for data access can be found here: http://www.bristol.ac.uk/alspac/researchers/access/. The ALSPAC study website contains details of all the data that are available (http://www.bristol.ac.uk/alspac/researchers/our-data/).
